# Effect of the Polydispersity of RBCs on the Recovery Rate of RBCs during the Removal of CPAs

**DOI:** 10.1155/2014/792302

**Published:** 2014-12-15

**Authors:** Heyuan Qiao, Weiping Ding, Yuncong Ma, Sijie Sun, Dayong Gao

**Affiliations:** ^1^Center for Biomedical Engineering, University of Science and Technology of China, Hefei, Anhui 230027, China; ^2^Department of Electronic Science and Technology, University of Science and Technology of China, Hefei, Anhui 230027, China; ^3^Department of Laboratory Medicine, University of Washington, Seattle, WA 98195, USA; ^4^Department of Mechanical Engineering, University of Washington, Seattle, WA 98195, USA

## Abstract

In the process of removing cryoprotectants from cryopreserved blood, the theoretically optimal operating condition, which is based on the assumption that the distribution of red blood cells is uniform, is often used to reduce or even avoid the hypotonic damage to cells. However, due to the polydispersity of cells, the optimal condition is actually not reliable. In this study, based on the discrete concept developed in our previous work, the effect of the polydispersity on the recovery rate of cells in the dilution-filtration system was statistically investigated by assigning three random parameters, isotonic cell volume, cell surface area, and osmotically inactive cell volume, to cells in small units of blood. The results show that, due to the polydispersity, the real recovery rate deviates from the ideal value that is based on uniform distribution. The deviation significantly increases with the standard errors of cell parameters, and it can be also magnified by high cryoprotectant concentrations. Under the effect of polydispersity, the uniform distribution-based optimized blood or diluent flow rate is not perfect. In practice, one should adopt a more conservative blood or diluent flow rate so that the hypotonic damage to cells can be further reduced.

## 1. Introduction

The process of removing cryoprotectants (CPAs) is one of five important steps in the cryopreservation of red blood cells (RBCs) [1–8], where the damage to RBCs could happen due to the cell volume excursion induced by osmotic disequilibria. In the past decades, many methods have been proposed to improve the process, such as centrifugation [9–12], dialysis [13–15], and dilution-filtration [16, 17].

To optimize the methods mentioned above, many theoretical models have also been developed [18–25]. In these models, almost without exception, the three parameters of RBCs, such as the cell volume at isotonic condition, the cell surface area, and the osmotically inactive cell volume, were all assumed to be identical. Thus, the mean values measured from experiments were applied. In the centrifugation method, a single cell was often studied to get optimal conditions for all cells. In the dialysis-based or dilution-filtration method, the blood was divided into discrete units so that the effect of the randomness of RBCs entering the washing system on the osmotic damage to RBCs and the washing time necessary to remove CPAs could be investigated; however, the cells were still considered the same [20, 24, 25]. In fact, the polydispersity of RBCs, that is, the difference between RBCs, exists widely [26]. For example, the RBCs have different sizes and shapes not only at different growth stages [27, 28], but also at the same stage due to health, gender, and ethnic differences [29, 30]. In abnormal subjects, the RBCs always have various differences compared to the ones in normal subjects (e.g., the volume distribution of RBCs would have a left shift peak in microcytic anemia patients or a right shift peak in pernicious anemia patients [31, 32]). In addition, the osmotically inactive cell volumes are also different in different growth stages for human RBCs [33–35], presenting in the form of random distributions [36, 37]. Therefore, the reliability of these models only using the mean properties of cells to some extent is questionable. In practice, the polydispersity of RBCs could cause the failure in not only the accurate prediction of the recovery rate of RBCs but also the optimization of the washing process of CPAs. Thus, it needs to be noticed in intensive study.

**Figure 1:**
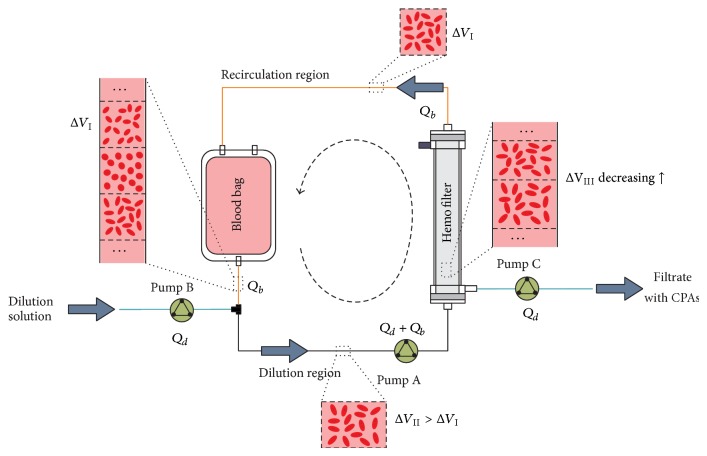
Schematic of removing CPAs with the diluent-concentration system.

In this work, the effect of the polydispersity of RBCs on the osmotic damage to RBCs or the recovery rate of RBCs in removing CPAs will be focused on. We will perform the study using the dilution-filtration system (Figure 1) in our previous work as an example to show the effect of polydispersity. The three characteristic parameters of RBCs as random variables will be integrated into the mass transfer equations. Then, the distribution of the cell maximum volumes under the polydispersity will be statistically analyzed, and the change of the distribution caused by the variation of the standard errors will be further studied. Finally, the effects of operating conditions, including cell swelling limits, cryoprotectant concentrations, and blood and diluent flow rates, will be discussed, and the safe operating condition under the effect of polydispersity will be given. To the best of our knowledge, the effect of the polydispersity of RBCs is for the first time considered in the cryopreservation of RBCs. Thus, our study could provide a strategy not only to predict more accurately the recovery rate of cells but also to search safer operating conditions for removing cryoprotectants. Furthermore, the thought in this work could be also extended to other washing systems to analyze the effect of the polydispersity of cells.

## 2. Modeling

### 2.1. Discrete Concept Revisited

In our previous work, a discrete concept was developed for the dialysis-based or dilution-filtration method to trace the volume variations of cells [15, 20, 24]. In the dilution-filtration method, the blood or red blood cell suspension to be washed is divided into a certain number of units. These units randomly enter the system, extend their volumes due to the dilution in the dilution region, restore their original volumes in the filter, go back to the blood bag, and wait to be selected into the system again (Figure 1). In the circulation of units, the CPA inside cells is transported out due to the decrease of the CPA outside cells induced by the dilution, filtered out of the hemofilter, and removed along with the filtrate, and the cells in units experience swelling and shrinkage.

The discretization makes it possible to trace the detailed information related to all units, including the volume of units, cell volumes, and solute concentrations inside and outside cells in units, to analyze whether the cells suffer osmotic damage and thus to statistically estimate the recovery rate of cells under a given operating condition. Compared to our previous work [17, 24, 25], the novelty of this work is that we consider the effect of the polydispersity of RBCs on the optimized operating condition for safely removing CPAs.

In the dilution-filtration system, the mass transfer of cryoprotectants from inside to outside cells in any unit can be unified and calculated separately. By means of a similar derivation method in the literature [24], the volume of RBCs and the intracellular concentration of CPAs can be calculated by the classic two-parameter model [40, 41] and the extracellular concentration of CPAs can be obtained according to mass conservation:

(1) dVcdt=Lp,cAcRTmn,1−mn,2+ms,1−ms,2 −Ps,cAcVs¯ms,1−ms,2

(2) dms,1dt=−1+Vs¯ms,1Vc−Vbc ×Ps,cAcms,1−ms,21+Vs¯ms,1+ms,1dVcdt

(3) dms,2dt=1+Vs¯ms,1ΔV−αVc ×αPs,cAc1+Vs¯ms,2ms,1−ms,2+αms,2dVcdt

(4) $$\begin{array}{c} m_{n,1}=\frac{m_{n,1}^{0}\left(V_{c}^{0}-V_{bc}-V_{s,1}^{0}\right)}{V_{c}-V_{bc}-V_{s,1}} \end{array}$$

(5) $$\begin{array}{c} m_{n,2}=\frac{m_{n,2}^{0}\left(\Delta V^{0}-\alpha V_{c}^{0}-V_{s,2}^{0}\right)}{\Delta V-\alpha V_{c}-V_{s,2}}, \end{array}$$

where *m*_*s*,1_ and *m*_*s*,2_ are CPA concentrations inside and outside RBCs, respectively (mol/kg H_2_O); *m*_*n*,1_ and *m*_*n*,2_ are NaCl concentrations inside and outside RBCs, respectively (mol/kg H_2_O); *V*_*s*,1_ and *V*_*s*,2_ are CPA volumes inside and outside RBCs, respectively (*μ*m^3^). *L*_*p*,*c*_ (m/Pa/s) and *P*_*s*,*c*_ (m/s) are hydraulic permeability and solute permeability of the RBC membrane, respectively. *V*_*c*_ is the RBC volume (*μ*m^3^), *A*_*c*_ is the cell membrane area (*μ*m^2^), and *V*_*bc*_ (*μ*m^3^) refers to the osmotically inactive volume of the cells at the isotonic condition (*V*_*c*_, *A*_*c*_, and *V*_*bc*_ are random variables). *R* is the universal gas constant (J/mol/K); *T* is the absolute temperature (K); $\bar{V_{s}}$ is the CPA partial molar volume (l/mol); and *α* is the cell density. Δ*V* is the volume of the blood units (it equals Δ*V*_*b*_ in the entrance region, Δ*V*_*b*_ + Δ*V*_*d*_ in the dilution region, and Δ*V*_*b*_ in the recirculation region and decreases from Δ*V*_*b*_ + Δ*V*_*d*_ to Δ*V*_*b*_ in the filter [24]). The superscript 0 denotes the previous time. The subscripts 1 and 2 denote inside and outside cells, respectively. The subscripts *b* and *d* denote blood and diluent, respectively. The subscripts *s* and *n* denote CPA and NaCl, respectively.

In the blood bag, the mixing occurs because the number of blood units is very large. Under this situation, cell volume and intracellular solute concentrations can be described by (1), (2), and (4) whereas extracellular solute concentrations can be calculated by the following equations approximately [24]:

(6) ms,2=∑ΔV0−αVc0−Vs,20ms,20     + αVc0−Vbc−Vs,10ms,10       ∑ΔV0−αVc0−Vs,20ms,20  −Vc−Vbc−Vs,1ms,1Vc0−Vbc−Vs,10   ×∑ΔVb−αVc−Vs,2−1,mn,2=∑(ΔVb0−αVc0−Vs,20)mn,20∑(ΔVb−αVc−Vs,2).

In the dilution-filtration system, at the dilution point (Figure 1), solute concentrations outside cells will change. Based on mass conservation, the relationships for them are as follows [24]:

(7) ΔVb−αVcI−Vs,2Ims,2I=ΔVb+ΔVd−αVcII−Vs,2IIms,2II,(ΔVb−αVcI−Vs,2I)mn,2I+ΔVdmn,2d  =ΔVb+ΔVd−αVcII−Vs,2IImn,2II,

where the superscripts *I* and *II* denote units before and after the dilution point, respectively, and *m*_*n*,2_^*d*^ is the NaCl concentration in the dilution solution.

### 2.2. Polydispersity of RBCs

Once the blood is divided into a certain number of units, we need to assign three parameters of cells for all units (here, we assume that cells are the same in one unit but different in different units). Then, we have to face two questions: the first is what distributions the isotonic volume of cells, the surface area of cells, and the osmotically inactive volume of cells statistically meet, respectively; the second is what the correlation among these parameters is. In practice, the osmotically inactive volume of cells cannot be directly measured and can be only obtained by extrapolation in hypotonic expansion experiments [33]. In this work, to simplify the topic studied, we assume that it is independent of the other two parameters. As for the isotonic volume and surface area of cells, we theoretically confirm that they are subject to bivariate normal distribution, shown in the following paragraphs.

RBCs usually differ from each other in size and shape. This fact results in the disproportion between the isotonic volume of cells (*V*_iso_) and the surface area of cells (*A*_*c*_). In experiments, the isotonic volume of cells and the surface area of cells are both subjected to normal distribution; however, they are associated with some certain relationship. If the shape of RBCs is spherical, they should rigorously be subjected to the 2/3 power relationship, but the shape of RBCs varies from sphere to oblate spheroid in different status. In the least squares analysis of volume versus area from the blood sample data of healthy adults, researchers concluded that they meet linear (with a correlation coefficient 0.969 in the literature [42] or 0.943 in the literature [43]) rather than 2/3 power relationship [42, 43].

**Figure 2:**
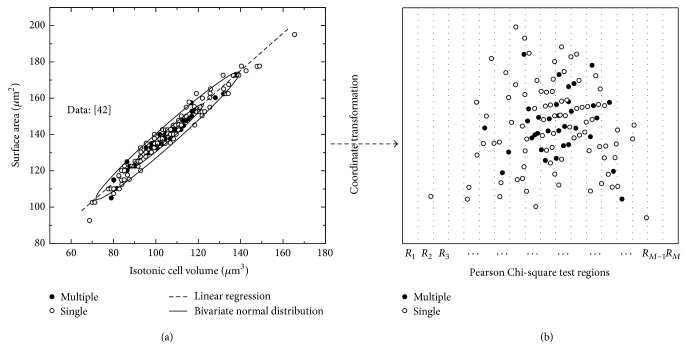
Relationship between isotonic cell volume and cell surface area (a) and Pearson Chi-square hypothesis test (b).

In our work, based on the pioneer work in the literature [39, 42], we assume that the joint distribution of the isotonic volume of cells and the surface area of cells is subjected to a bivariate normal distribution, as shown in Figure 2(a). The distribution parameters including mean value (*μ*_*v*_ and *μ*_*a*_), standard error (*σ*_*v*_ and *σ*_*a*_), and correlation coefficient (*r*) are estimated by maximum likelihood estimation, which are presented below (the subscripts *v* and *a* indicate isotonic cell volume and surface area, resp.):

(8a) $$\begin{array}{c} \mu_{v}=\frac{\sum_{i=1}^{N}v_{i}}{N} \end{array}$$

(8b) $$\begin{array}{c} \mu_{a}=\frac{\sum_{i=1}^{N}a_{i}}{N} \end{array}$$

(8c) $$\begin{array}{c} \sigma_{v}^{2}=\frac{\sum_{i=1}^{N}{\left(v_{i}-\mu_{v}\right)}^{2}}{N} \end{array}$$

(8d) $$\begin{array}{c} \sigma_{a}^{2}=\frac{\sum_{i=1}^{N}{\left(a_{i}-\mu_{a}\right)}^{2}}{N} \end{array}$$

(8e) $$\begin{array}{c} r=\frac{\sum_{i=1}^{N}\left(v_{i}-\mu_{v}\right)\left(a_{i}-\mu_{a}\right)}{\sqrt{\sum_{i=1}^{N}{\left(v_{i}-\mu_{v}\right)}^{2}}\sqrt{\sum_{i=1}^{N}{\left(a_{i}-\mu_{a}\right)}^{2}}}, \end{array}$$

where *N* is the number of observations. Then, the joint distribution probability density function (PDF) can be described in vector format as below:

(9) $$\begin{array}{c} f\left(X\right)=\frac{1}{2\pi \sqrt{\left|\Sigma \right|}}\exp\left[-\frac{1}{2}{\left(X-\mu \right)}^{T}\Sigma^{-1}\left(X-\mu \right)\right], \end{array}$$

where $X=\left[\begin{array}{c} v\\ a \end{array}\right]$ is the parameter vector, $\mu =\left[\begin{array}{c} \mu_{v}\\ \mu_{a} \end{array}\right]$ represents the mean vector, and $\Sigma =\left[\begin{array}{cc} \sigma_{v}^{2} & r\sigma_{v}\sigma_{a}\\ r\sigma_{v}\sigma_{a} & \sigma_{a}^{2} \end{array}\right]$ represents the covariance matrix.

In this work, the Pearson Chi-square test of goodness of fit is applied for hypothesis test of our bivariate normal distribution [44]. The null hypothesis is that the isotonic cell volume and surface area are subjected to bivariate normal distribution at 5% significance level.

First, we perform coordinate transformation for region division and frequency statistics. The inverse matrix of the covariance matrix (Σ^−1^) is diagonalized by orthogonal matrix *V*:

(10) $$\begin{array}{c} V^{T}\Sigma^{-1}V=D,\;D=\left[\begin{array}{cc} \lambda_{1} & 0\\ 0 & \lambda_{2} \end{array}\right], \end{array}$$

where *D* is the diagonalized covariance matrix. If we introduce $Y=\left[\begin{array}{c} v^{\prime }\\ a^{\prime } \end{array}\right]$ and set *X* = *VY* + *μ*, then we can get

(11) fY=12πΣexp⁡−12YTDY=λ1λ22πexp⁡−12λ1v′2+λ2a′2.

To calculate the Pearson statistic *χ* ^2^ = ∑_*j*=1_^*M*^((*n*_*j*_ − *NP*_*j*_)^2^/*NP*_*j*_), we divide the (*v*′, *a*′) plane into *M* mutually disjoint regions *R*_1_ ⋯ *R*_*M*_ (*n*_*j*_ is the frequency statistics in each region, and *P*_*j*_ is the theoretical probability in each region). *R*_1_ ⋯ *R*_*M*_ can be chosen as finite or infinite rectangle to simplify the calculation. Here, we use a serial of equidistant lines parallel to *y*-axis to divide the (*v*′, *a*′) plane into different regions, which is shown in Figure 2(b) (the multiple points indicate that a same value of isotonic cell volume and surface area is observed multiple times (usually ≥3) in the measurement).

Under the coordinate transformation, *n*_*j*_ is counted in each region (multiple points counted as 3) and *P*_*j*_ is calculated by

(12) Pj=∬Rjf(Y)dY=∫hj−1hj∫−∞∞λ1λ22πexp⁡−12λ1v′2+λ2a′2dv′da′=∫hj−1hjλ12πexp⁡⁡−12λ1v′2dv′ ·∫−∞∞λ22πexp⁡⁡−12λ2a′2da′=Φhjλ1−Φhj−1λ1,

where *Ф*(*x*) represents the cumulative distribution function of the standard normal distribution.

Then, we can finally get the Chi-square critical value (the degree of freedom *d* is calculated by the expression *d* = *M* − *L* − 1. In this study, *M* = 13 is the number of different regions divided from the (*v*′, *a*′) plane in Pearson Chi-square test and  *L* = 5 is the number of estimated parameters):

(13) $$\begin{array}{c} \chi^{2}=\overset{M}{\underset{j=1}{\sum }}\frac{{\left(n_{j}-NP_{j}\right)}^{2}}{NP_{j}}<\chi_{0.05}^{2}\left(d\right). \end{array}$$

The null hypothesis cannot be rejected at the 5% significance level and, thus, the isotonic cell volume and surface area are subjected to bivariate normal distribution with the estimated parameters. Therefore, the Pearson Chi-square test proves that the bivariate normal distribution is more suitable than the linear regression.

As for the osmotically inactive cell volume (*V*_*bc*_), though it may depend on the cell stage or cell aging, there are not any reliable data or literature to show its direct connections with the other two parameters. Here an independent normal-distributed random parameter is assumed in this work. The distribution of *V*_*bc*_ is shown as follows:

(14) $$\begin{array}{c} \mu_{vbc}=\frac{\sum_{i=1}^{N}v_{bc_{i}}}{N},\;\;\sigma_{vbc}^{2}=\frac{\sum_{i=1}^{N}{\left(v_{bc_{i}}-\mu_{vbc}\right)}^{2}}{N},\\ f\left(v_{bc}\right)=\frac{1}{\sqrt{2\pi }\sigma_{vbc}}\exp\left[\frac{{\left(v_{bc}-\mu_{vbc}\right)}^{2}}{2\sigma_{vbc}^{2}}\right], \end{array}$$

where the mean value (*μ*_*vbc*_) and standard error (*σ*_*vbc*_) are derived from the several groups of fitted values listed in the literature [33].

### 2.3. Simulation Strategy

The purpose of this work is to theoretically and statistically study the effect of the polydispersity of RBCs in the process of removing cryoprotectants. The strategy is as follows. Firstly, the blood is divided into a certain number of small units, which are traced and controlled by a program code to randomly enter the washing system. Secondly, the three parameters of RBCs are generated by another program code and then randomly assigned to all units. Then, the above two-parameter equations are used to calculate the volume variations of cells and the concentration variations of CPAs in all units. Finally, the effect of the polydispersity on the recovery rate of RBCs under various situations is studied statistically.

In the simulation, the cryoprotectant was glycerol, the diluent was the isotonic solution only containing NaCl (290 mOsm), and the blood was mimicked by the red blood cell suspension only containing NaCl and glycerol (NaCl: 290 mOsm; glycerol: 6500 mOsm; and hematocrit: 30%). In the dilution-filtration system [17], the lumen volume of the plasma filter (Plasmflo AP-05H/L, ASAHI Co., Japan), the dilution region volume, and the recirculation region volume were 85 mL, 5 mL, and 10 mL, respectively (the diameter of the tubing was 4 mm). Other parameters used are listed in Table 1.

In this work, 200 mL of blood was divided into 5000 units so that the error caused by the division was less than 0.5% (the volume of units was 40 *μ*L and cells were considered identical in one unit but different in different units). Five thousand sets of random parameters (i.e., isotonic cell volume, cell surface area, and osmotically inactive volume) were generated by a program code and assigned to cells in 5000 units, subjecting to the distributions mentioned above. In calculation, units were randomly selected to enter the system and the cell volume changes in all units were traced, using another program code. If the cell maximum volume in one unit exceeds the upper tolerance limit (here, the upper tolerance limit for completely avoiding the hypotonic damage was set to 1.53 *V*_iso_ according to the literature [45]), all cells in this unit will be marked and regarded as dead. Then, the recovery rate of cells was statistically obtained. In this study, the program codes, developed in FORTRAN, were used to simulate the washing process and perform the statistical analysis. For each operating condition, the simulation was repeated 6 times and a mean value was used.

## 3. Results and Discussion

### 3.1. Statistical Distribution of the Maximum Volumes of the RBCs

**Figure 3:**
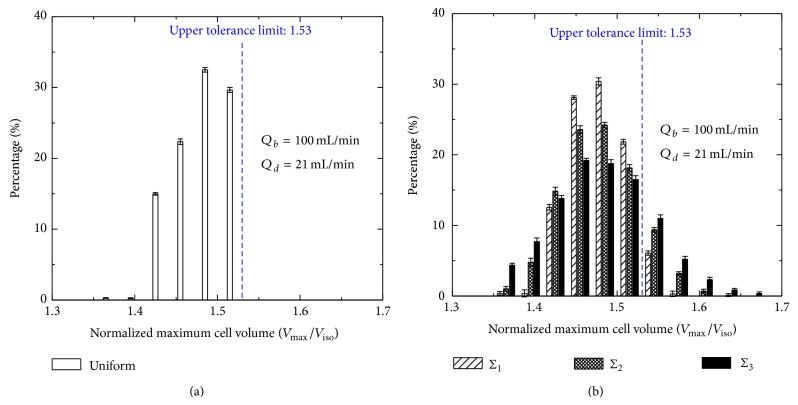
The comparison between cell maximum volume distributions under uniform (a) and random (b) parameters of cells.

**Figure 4:**
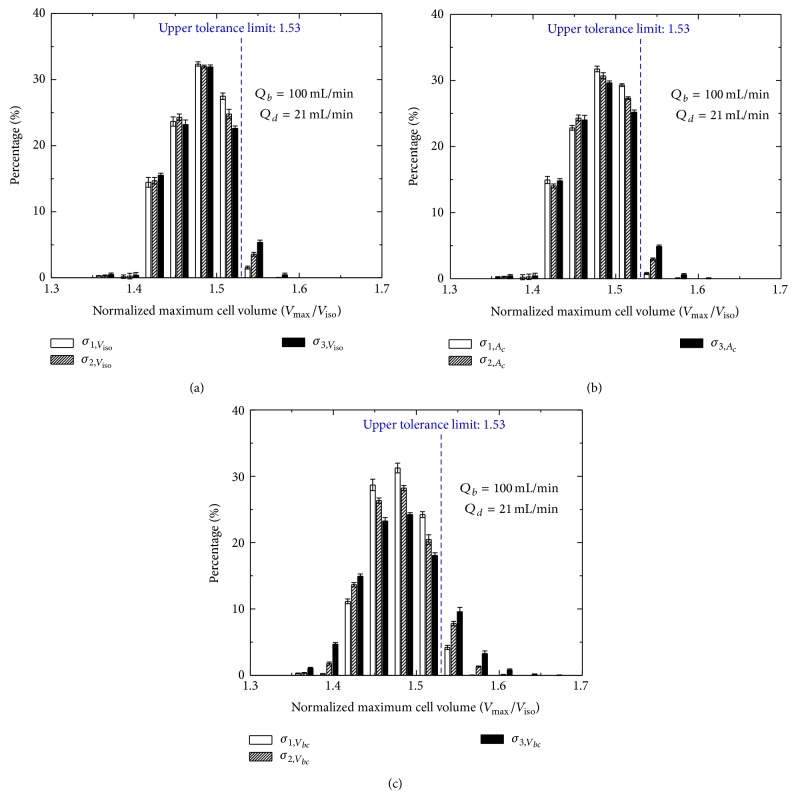
The effects of standard errors of isotonic cell volume (a), cell surface area (b), and osmotically inactive cell volume (c) on distributions of cell maximum volumes.

In the dilution-filtration system, if all cells are assumed to be the same (i.e., the parameter distributions of cells are assumed to be uniform), when the optimized diluent flow rate is used, the maximum volumes of all cells are below the upper tolerance limit and the hypotonic damage to cells can be avoided completely (Figure 3(a)). However, due to the effect of the cell polydispersity, there are still some cells suffering from the hypotonic damage in practice (Figure 3(b); the standard errors of cell parameters are listed in Table 2). Moreover, the damage will increase with the standard error of no matter isotonic volume, surface area, or inactive volume (Figure 4 and Table 3). The effect of the osmotically inactive cell volume is relatively more significant. In addition, our results also show that the distribution of the cell maximum volume is normal under the condition of the polydispersity (Figure 4). Here, the optimized diluent flow rate is an allowable value set by system, which makes the maximum volume of the RBCs very close to the upper tolerance limit and is kept constant in all cycles.

**Figure 5:**
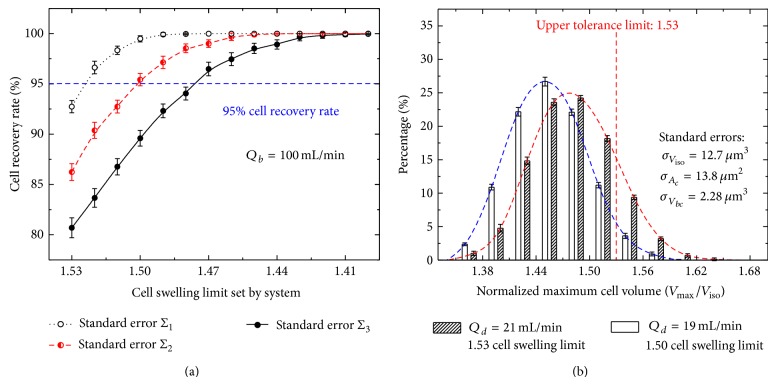
The comparisons of cell recovery rates (a) and cell maximum volume distributions (b) under various cell swelling limits set by system.

Having established that the polydispersity results in the optimized condition deviating from the actual expectation, we wonder how to improve the condition to minimize the effect of the polydispersity. For the dilution-filtration system, an effective method is to set a small or conservative cell-swelling limit in searching the optimized diluent flow rate (Figure 5(a)). By doing so, the distribution of the cell maximum volume will move left, as shown in Figure 5(b), and the recovery rate of cells will increase. For example, when the blood flow rate is 100 mL/min, if the three standard errors are 12.7 *μ*m^3^, 13.8 *μ*m^2^, and 2.28 *μ*m^3^, respectively, and the expected recovery rate is higher than 95%, the cell swelling limit should be smaller than 1.50 *V*_iso_, instead of 1.53 *V*_iso_ (Figure 5(a)). Corresponding to the change, the optimized diluent flow rate should be smaller than 19 mL/min (Figure 5(b)).

### 3.2. Effects of the RBC Polydispersity under Various Initial CPA Concentrations

**Figure 6:**
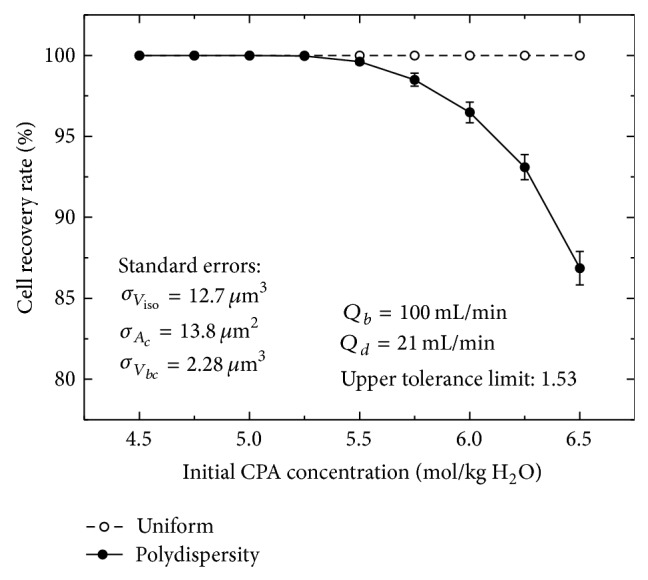
The effect of the cell polydispersity on the recovery rate of cells under various initial CPA concentrations.

Since the polydispersity can cause the decrease in the recovery rate of cells, even if the optimized condition is used, it is important to understand in practice when the effect of the polydispersity becomes significant. Our results show that the polydispersity decreases the recovery rates of cells, especially when the initial CPA concentration is high (the high concentration of CPAs causes the large volume change of cells in cycles and then the effect of the polydispersity is accumulated). Moreover, the difference of the recovery rates of cells between uniform and random distributions also increases with the initial CPA concentration (Figure 6). For example, when the three standard errors are 12.7 *μ*m^3^, 13.8 *μ*m^2^, and 2.28 *μ*m^3^, respectively, if the CPA concentration added to blood at the beginning of cryopreservation is increased from 5.5 mol/kg H_2_O to 6.5 mol/kg H_2_O, the difference will increase from 0.38% to 13.14%. Therefore, a more conservative cell-swelling limit should be set for the dilution-filtration system in practice when a higher CPA concentration needs to be removed.

### 3.3. Effects of the RBC Polydispersity under Various Blood and Diluent Flow Rates

**Figure 7:**
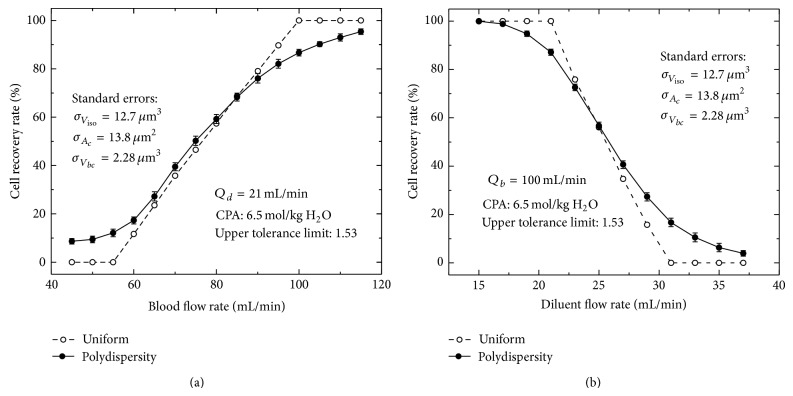
The effect of the cell polydispersity on the recovery rate of cells under various blood flow rates (a) or diluent flow rates (b).

**Figure 8:**
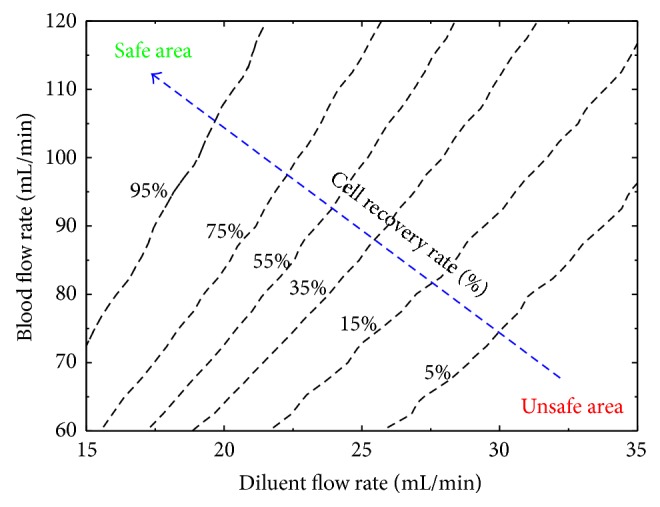
The recovery rates of cells under various blood and diluent flow rates.

In the dilution-filtration system, diluent and blood flow rates are two controllable factors to reduce the hypotonic damage to cells. Under the assumption of uniform distribution, for a fixed diluent flow rate, the ideal recovery rate of cells first remains 0, then increases approximately linearly, and finally keeps 1 as the blood flow rate increases (Figure 7(a); the upper inflection point is called the uniform distribution-based optimal blood flow rate for a fixed diluent flow rate). However, due to the polydispersity, the change trend of the real recovery rate of cells presents a smooth curve rather than a broken line: when the blood flow rate is larger, the real recovery rate is less than the ideal one; when the blood flow rate is lower, the result is just the opposite (Figure 7). Similar to the effect of polydispersity under various blood flow rates, the deviation between ideal and real recovery rates of cells also exists under various diluent flow rates (Figure 7(b); the lower inflection point is called the uniform distribution-based optimal diluent flow rate for a fixed blood flow rate). Our results indicate that the uniform distribution-based optimal blood or diluent flow rate is inappropriate and can still cause cell loss because of the polydispersity of cells. Therefore, in practice, to reduce the effect of the polydispersity of cells, a more conservative condition (a higher blood flow rate or a lower diluent flow rate) should be used so that the expected recovery rate of cells can be obtained. In this work, a basic picture on the recovery rates of cells under various blood and diluent flow rates is shown in Figure 8, taking into account the polydispersity of cells. The picture clearly shows the nonlinear trend of the recovery rate of cells for a fixed blood or diluent flow rate and it can be used to practically guide the washing process of CPAs using the dilution-filtration system.

## 4. Conclusions

In this work, we provide a strategy to statistically study the effect of the polydispersity of RBCs on the recovery rate of RBCs in removing CPAs from cryopreserved blood by assigning three random parameters to all cells, isotonic volume, surface area, and inactive volume. To confirm the effect of the polydispersity, the deviation between ideal and real recovery rates of RBCs in the dilution-filtration system proposed in our previous work is investigated. The results show that due to the polydispersity, the real recovery rate deviates from the ideal one; moreover, the deviation significantly increases with the standard errors of cell parameters (if the three standard errors vary from 6.35 *μ*m^3^, 6.9 *μ*m^2^, and 1.14 *μ*m^3^ to 19.05 *μ*m^3^, 20.7 *μ*m^2^, and 3.42 *μ*m^3^, resp., the deviation will increase from 6.36% to 19.72%). The high concentration of CPAs added at the beginning of the cryopreservation process can magnify the effect of polydispersity. If the concentration is increased from 5.5 mol/kg H_2_O to 6.5 mol/kg H_2_O, when the three standard errors are 12.7 *μ*m^3^, 13.8 *μ*m^2^, and 2.28 *μ*m^3^, respectively, the deviation will increase from 0.38% to 13.14%. Under the effect of polydispersity, the uniform distribution-based optimized blood or diluent flow rate is not perfect. In practice, one still needs to adopt a more conservative condition, that is, a blood flow rate higher than the optimized blood flow rate or a diluent flow rate lower than the optimized diluent flow rate, so that the expected recovery rate of cells can be obtained.

In this study, we only theoretically study the effect of the polydispersity of RBCs. To validate the simulation with the measured recovery rate of RBCs, one should first experimentally determine the distributions of the three parameters of RBCs, then measure the recovery rate of RBCs after using the dilution-filtration system under a given operating condition, and finally compare the measured recovery rate of RBCs with the theoretical value. In practice, to establish a customized filtration-dilution system by considering the polydispersity of RBCs as well as the associated optimal blood and diluent flow rates, an extra storage for the table that includes the precalculated optimal operating conditions needs to be embedded into the system. In addition, it should be noted that the strategy developed here could be used for not only the cryoprotectant unloading process but also the cryoprotectant loading process.

## Figures and Tables

**Table 1:** Parameters used in this paper.

Parameters	Units	Values	Reference
Isotonic RBC volume, *V*_iso_	*μ*m^3^	89.8 ± 12.7^*^	[33]
RBC surface area, *A*_*c*_	*μ*m^2^	134.1 ± 13.8^*^	[33]
Osmotically inactive RBC volume, *V*_*bc*_	*μ*m^3^	39.4 ± 2.28^*^	[33]
Hydraulic permeability, *L*_*p*,*c*_	m/Pa/s	1.74 × 10^−12^	[38]
Glycerol permeability, *P*_*s*,*c*_	m/s	6.61 × 10^−8^	[38]
Correlation coefficient, *r*	/	0.943	[39]
Absolute temperature, *T*	K	298	

^*^ *V*_iso_, *A*_*c*_, and *V*_*bc*_ are random variables (mean ± STD).

**Table 2:** Standard errors of cell parameters used in the calculation.

Standard error	Group 1	Group 2	Group 3
*σ*_*i*,*V*_iso__	6.35 *μ*m^3^	12.7 *μ*m^3^	19.05 *μ*m^3^
*σ*_*i*,*A*_*c*__	6.9 *μ*m^2^	13.8 *μ*m^2^	20.7 *μ*m^2^
*σ*_*i*,*V*_*bc*__	1.14 *μ*m^3^	2.28 *μ*m^3^	3.42 *μ*m^3^
∑_*i*_	{*σ*_1,*V*_iso__, *σ*_1,*A*_*c*__, *σ*_1,*V*_*bc*__}	{*σ*_2,*V*_iso__, *σ*_2,*A*_*c*__, *σ*_2,*V*_*bc*__}	{*σ*_3,*V*_iso__, *σ*_3,*A*_*c*__, *σ*_3,*V*_*bc*__}

**Table 3:** Cell mortality rates under different standard errors.

Standard error	Group 1	Group 2	Group 3
*σ*_*i*,*V*_iso__	0.78%	3.06%	5.54%
*σ*_*i*,*A*_*c*__	1.56%	3.62%	5.82%
*σ*_*i*,*V*_*bc*__	4.22%	9.20%	13.79%
∑_*i*_	6.36%	13.14%	19.72%
